# A Retrospective Comparative Study of Long-Term Outcomes Following Cervical Total Disc Replacement Versus Anterior Cervical Discectomy and Fusion

**DOI:** 10.7759/cureus.32399

**Published:** 2022-12-11

**Authors:** Kelechi Eseonu, Edward Laurent, Habeeb Bishi, Hassan Raja, Kuppuswamy Ravi, Zaher Dannawi

**Affiliations:** 1 Spine Surgery, Guy's and St Thomas' NHS Foundation Trust, London, GBR; 2 Trauma and Orthopaedics, Mid and South Essex NHS Foundation Trust, Basildon, GBR; 3 Trauma and Orthopaedics, The Shrewsbury and Telford Hospital NHS Trust, Telford, GBR; 4 Trauma and Orthopaedics, Barts Health NHS Trust, London, GBR; 5 Trauma and Orthopaedics, Mid and South Essex NHS Foundation Trust, London, GBR; 6 Spine Surgery, Mid and South Essex NHS Foundation Trust, London, GBR

**Keywords:** degenerative disc disease, visual analogue scale (vas), neck disability index (ndi), cervical spine, total disc replacement (tdr), anterior cervical discectomy and fusion (acdf)

## Abstract

Introduction

The traditional treatment for patients with radiculopathy and myelopathy caused by degenerative disc disease was anterior cervical discectomy and fusion (ACDF). However, a documented complication of ACDF is adjacent segment degeneration (ASD). An alternative that was developed was total disc replacement (TDR).

The aim of this study was to determine and compare the short- and medium-to-long-term outcomes after a TDR or ACDF.

Methods

A retrospective review of 154 patients who had single and two-level ACDFs and 90 TDRs performed by a single surgeon between 2011 and 2017 was conducted. Parameters for comparisons include both radiological evaluation and patient-reported outcome measures (PROMS) at six weeks, one year, and two years postoperatively. The Neck Disability Index (NDI) and the visual analogue scale (VAS) for neck and arm pain are used to evaluate pain, function, patient satisfaction, and overall clinical success.

Results

TDR and ACDF showed significant improvement in NDI and VAS when compared to pre- and post-operatively at both six weeks (p<0.05 & P=0.032, respectively) and two years (p<0.05 & 0=0.026, respectively). TDR vs. ACDF showed no significant difference (p<0.05). VAS scores after ACDF showed improvement from 13.41 to 3.94 at two years (p<0.001). TDR showed similar scores of 12.5 to 3.55 (p<0.001). The radiological fusion rate at 12 or 24 months showed no significant difference between the two groups. There were two cases that required re-operation after ACDF (1.2%), and two that required TDR (2.2%).

Conclusion

Both TDR and ACDF lead to clinically significant improvements in pain and function scores. We did not find a statistically significant difference in NDI and VAS in the neck and arm.

The results are in agreement with others' assessments of these two treatment modalities. Our conclusions supplement the literature about these operative options for degenerative disc disease of the cervical spine and are a useful addition to the armamentarium in the assessment of patients with degenerative pathology of the c-spine.

## Introduction

Since its development by Smith-Robinson and Cloward in the 1950s, the traditional treatment for patients with radiculopathy and myelopathy due to degenerative disc disease has been anterior cervical discectomy and fusion (ACDF) [1]. A well-documented complication of this procedure is adjacent segment degeneration (ASD), with studies suggesting up to 25% of patients develop this within 10 years [2]. This diagnosis is made when there is myelopathy or radiculopathy at the adjacent level and can lead to re-operation at the affected level.

Cervical total disc replacement (TDR) was initially introduced in the 1960s with limited success due to complications including instability and prosthesis subsidence [3]. Renewed interest in TDR has been associated with the development of metal-on-metal and metal-on-polyethylene discs, and a number of these have been licenced by the United States Food and Drug Administration (FDA) [4].

Since an increase in their use in the 1990s, TDR has become an alternative to ACDF whilst maintaining motion at the affected level. Fusion at one level can lead to a compensatory increase in movement and stress at adjacent levels, but the exact mechanism of adjacent segment disease is poorly understood. It is postulated that retention of motion in a cervical segment can reduce the rate of adjacent segment disease [5]. At present, it is unclear whether ASD following ACDF surgery occurs due to segmental fusion or normal physiological spine deterioration [6]. The evidence on the medium- and long-term benefits of TDR, when compared with ACDF, is varied.

The aim of this study was to compare the short- and medium-term outcomes in adult patients (>18 years of age) undergoing total disc replacement (TDR) or ACDF for cervical myelopathy or radiculopathy secondary to degenerative disc disease, as well as the incidence and causes of reoperation after both procedures.

## Materials and methods

Patient selection

We performed a retrospective review of prospectively collected data on 154 patients following single- and two-level ACDFs and 90 single- and two-level TDRs performed by a single surgeon between 2011 and 2017 with at least a two-year follow-up. The inclusion and exclusion criteria are detailed in Table 1.

**Table 1:** Inclusion and exclusion criteria of the case study DEXA: dual-energy X-ray absorptiometry

Inclusion criteria	Exclusion criteria
Cervical disc herniation	Developmental cervical stenosis
Degenerative cervical spinal stenosis	Ossification of the posterior longitudinal ligament
Conservative treatment for at least three months	Unstable cervical spine with angular displacement > 2° or vertical displacement > 2 mm
Age >=18 years	Osteoporosis diagnosed by DEXA scan or with previous spinal compression fractures
	Congenital cervical abnormalities (e.g., Klippel-Feil syndrome)
	Cervical spinal infection
	Ankylosing spondylitis
	A history of cervical spine surgery

Surgical procedure

The surgery was performed using a conventional left-sided anterior approach to the cervical spine. The symptomatic disc and then the posterior longitudinal ligament (PLL) were excised. In the ACDF group, the Cervios Synthes cage with local bone was implanted into the intervertebral space. In the TDR group, the Prodisc-C VIVO prosthesis was implanted after accurate measurement.

Both groups of patients underwent statistical analysis using the Statistical Package for the Social Sciences (SPSS) for Windows version 18 (IBM SPSS Inc., New York, USA). Comparisons of neck disability index (NDI) and visual analogue scale (VAS) scores were performed using a Student's t-test with differences of p<0.05 considered significant.

Postoperative care

All patients received a standard rehabilitation program, which is aimed at returning them to their normal activities as quickly as possible. A cervical collar was applied to all patients until six weeks' follow-up. Both groups were asked to avoid non-steroidal anti-inflammatory drugs (NSAIDs) until three months post-surgery.

Radiographic assessment

Radiographic parameters were collected by screening neutral and dynamic flexion-extension lateral radiographs during each follow-up examination.

Cervical lordosis was determined by measuring Cobb’s angle between the inferior endplates of C2 and C7 vertebral bodies in the lateral cervical spine X-ray. Radiographic success in the ACDF group was equated with successful fusion, which is defined as the following: less than 2° of angular motion in flexion and extension; evidence of bridging bone across the disc space; and radiolucent lines at no more than 50% of the graft vertebral interfaces. Radiographic success in the TDR group was defined as at least 2° of angular motion in flexion or extension or no evidence of bridging trabecular bone across the disc space.

All radiological outcomes were reviewed by a spinal surgeon and a musculoskeletal radiologist who were blinded to the treatment regimen, using the Picture Archiving and Communications System (PACS) software and a PACS workstation (Centricity 2.0, General Electric Medical Systems, Milwaukee, WI).

Outcome assessment 

All patients were required to return to the clinic to assess the Patient Reported Outcome Measures (PROMS) as well as radiological evaluation at six weeks, one year, and two years post-operatively, with a further clinical review via telephone consultation in December 2019. Outcome measures used to evaluate pain, function, and patient satisfaction included the Neck Disability Index (NDI) [7] and the visual analogue scale (VAS) for neck and arm pain [8].

Revision surgery was defined as any secondary procedure at an index-level segment and was classified as a removal, revision, supplemental fixation, or reoperation. Adjacent-level subsequent surgeries were documented for further analysis.

"Overall procedure success" after either procedure was determined by a composite endpoint of variables [9]. These include a greater than 15-point improvement in the NDI score; maintenance of improvement in neurological status postoperatively; no serious adverse side effects related to the implant or surgical procedure; and no requirement for revision surgery.

## Results

Patient population

Baseline demographic characteristics were evenly matched across each group (Table 2). There was no statistically significant difference in age (52.8 ACDF vs. 49.1 TDR; p > 0.05), gender distribution (48:52, male: female in the ACDF group vs. 49:51, male: female in the TDR group; p > 0.05), or length of follow-up between the two groups (the average in the ACDF group was 63 months (range 24-96 months) compared with 57 months (24-90 months) in the TDR group). In addition, there was no statistical difference in the two-year follow-up rate between the two groups (93.3% ACDF vs. 94.6% TDR group, p > 0.05).

**Table 2:** Baseline data of the study population ACDF: anterior cervical decompression and fusion; TDR: total disc replacement; f/u: follow up

	ACDF			TDR
	One level	Two level	ACDF (total)	
Age (average)	51.4	54.6	52.8	49.4
Number of patients	86	68	154	90
Two-year f/u (%)	93.3			94.6
F/U (yrs. {range})	5.15 (2-8)			4.7 (2-7.6)

Neck disability index (NDI) 

At the six-week follow-up, the mean Neck Disability Index (NDI) showed statistically significant improvements between pre-op and post-op in both the TDR (p<0.05) and ACDF groups (p=0.032). Statistical improvement continued at the two-year follow-up in both the TDR (p<0.05) and ACDF groups (p=0.026) (Figure 3).

When comparing TDR against ACDF, in terms of NDI, there was no statistically significant difference.

**Figure 1:**
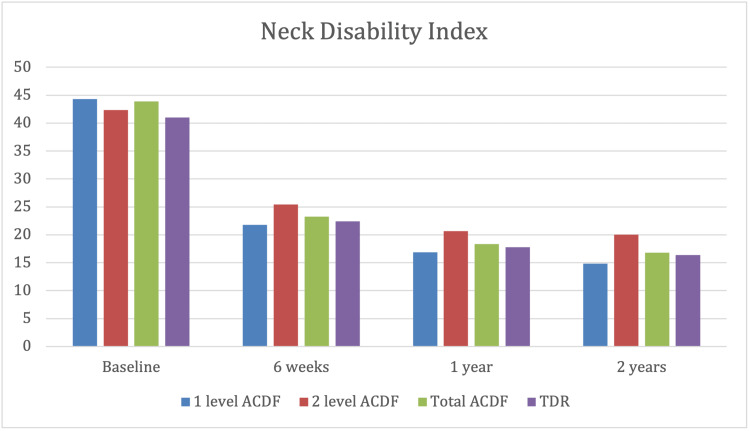
Comparison of the Neck Disability Index (NDI) score between the anterior cervical decompression and fusion (ACDF) group and the total disc replacement (TDR) group by the Student's t-test

Further analysis compared the single-level and two-level ACDF NDI outcomes. There were 86 single-level and 68 two-level ACDFs in the cohort. There was no statistically significant difference in age, gender, or length of follow-up between the two groups. Both groups showed statistically significant improvement from pre-op to six weeks and at one and two years of follow-up. In the single-level group, NDI fell from 44.3 pre-operatively to 21.7, 16.9, and 14.8 at six weeks, one-year, and two-year follow-up, respectively (an improvement of 50.4%, 61.5%, and 66.3%). In the two-level group, NDI fell from 42.3 to 25.4, and 20.7 to 20.0 at six weeks, one year, and two years, respectively (an improvement of 39.9%, 51.3%, and 52.7%). At two years' follow-up, a comparison of these two sub-groups revealed a significant improvement in ACDF at the single level when compared to the two-level ACDF procedure (p = 0.023) (Figure 1)

Neck and arm pain VAS scores

**Figure 2:**
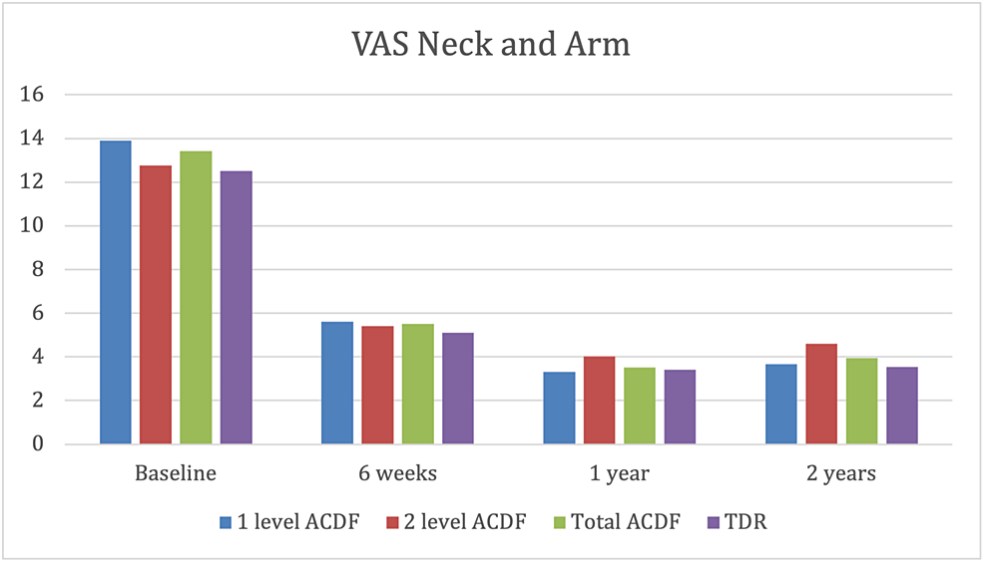
Comparison of visual analogue scale (VAS) neck and arm score between anterior cervical decompression and fusion (ACDF) group and total disc replacement (TDR) group by the Student's t-test

The TDR and the ACDF group had similar VAS neck and arm pain scores at baseline, and both demonstrated significant improvement from baseline to the two-year review (13.41 to 3.94 in the ACDF group (p<0.001) and 12.41 to 3.82 in the TDR (p<0.001)). In addition, there was no statistically significant difference in VAS head and neck improvement between the ACDF and TDR groups (p>0.05) (Figure 2).

Radiological assessment

There was no statistically significant difference in the "radiological success rate" (as defined in the methods section) at 12 or 24 months in the TDR versus the ACDF group. No cases of implant migration or subsidence that required operative intervention were reported during the follow-up period in either cohort.

Complication rate

In the ACDF group, there were two cases that required reoperation (1.2%); one due to adjacent segment degeneration identified clinically and radiographically at 24-month follow-up, and one due to persistent myelopathic symptoms after the index procedure, which required posterior decompression (laminectomy) and stabilisation (Table 3).

Two cases of reoperation were identified in the TDR group (2.2%) and were performed two and five years after the index procedure due to adjacent segment degeneration. There was no statistically significant difference in the rate of reoperation by the final follow-up when comparing the TDR and the ACDF groups (p>0.05) (Table 3).

**Table 3:** Summary of cases requiring reoperation ACDF: anterior cervical decompression and fusion; TDR: total disc replacement; ASD: adjacent segment disease; NDI: neck disability score; VAS: visual analogue scale; F: female; M: male

	Case one	Case two	Case three	Case four
Sex	F	F	M	F
Age	41	63	23	45
Primary procedure	ACDF	ACDF	TDR	TDR
Index implant level	C6/7	C5/6	C6/7	C4/5
Reoperation level	C5/6	C5/6	C5/6	C5/6
Interval between primary and secondary treatment	24 months	Nine months	62 months	14 months
Reoperation	ACDF due to ASD	Posterior decompression and fixation due to ongoing myelopathy	TDR	ACDF due to ASD
Outcome	Good clinical outcome with an improvement of NDI and VAS	Excellent clinical outcome at six months post the second procedure	Good clinical outcome with an improvement of NDI and VAS	Good clinical outcome with an improvement of NDI and VAS

In addition, there was a single intraoperative dural tear and a case of Horner's syndrome in the ACDF group, which recovered completely by two weeks postoperatively. There was one post-operative Brown-Sequard syndrome in the TDR group that had an excellent recovery by the six-week review. There was no significant difference in the overall complication rate between the two groups (p>0.05) (Table 4).

**Table 4:** Summary of complications ACDF: anterior cervical decompression and fusion; TDR: total disc replacement; M: male; F: female

	Sex	Age	Primary procedure	Index level	Complication details	Outcome
Case one	M	?	ACDF	?	Horner’s syndrome	Excellent recovery by two weeks post index procedure
Case two	F	61	ACDF	C6/7	Dural tear requiring intra-operative repair	Excellent recovery by two weeks post index procedure
Case three	M	53	TDR	C7/T1	Partial Brown Sequard syndrome	Significant recovery by six weeks

Overall procedure success

The "overall procedure success" (as defined in the method section) showed the ACDF group to be 90.1% successful, with the TDR group being 92.1% successful. There was therefore no statistically significant difference between the two groups (p>0.05).

## Discussion

The results of our retrospective single-surgeon study compared statistically equivalent cohorts of patients undergoing single or two-level ACDFs and TDR. ACDF has been widely used and proven to clinically provide stability for cervical degenerative disc disease, especially cervical radiculopathy and spondylotic myelopathy. However, it has been suggested that ACDF may alter the rotational movement of the cervical spine axis and increase the load on adjacent segments, thereby hastening the degeneration of adjacent segments [10, 11]. Early clinical improvement is achieved by complete surgical decompression, removal of pathologically herniated discs, and recovery of pathological spinal cord and nerve roots.

In our study, the postoperative neck disability index (NDI) and VAS scores of both the ACDF group and the TDR group were significantly improved from their preoperative scores, and there was no significant difference between the two groups at different time points (up to at least a two-year follow-up). Published randomised controlled clinical studies have shown the non-inferiority of short-term outcomes of TDR surgery compared to ACDF surgery [12-14]. We similarly did not find a significant difference in overall complication rates due to adjacent segment degeneration between the two procedures at a mean of 5.4 years of follow-up across both groups.

Veeravagu et al. [15] reviewed 28,777 cases undergoing ACDF and found that 9.13% of the single-level and 10.7% of the multilevel ACDF groups required a second operation within two years. They reported that the number of fusion levels was significantly correlated with the rate of reoperations. At 48 months, Davis et al. [16] reported a cumulative incidence of 4% reoperation at the index level in the TDR group. We report no cases of pseudoarthrosis, migration, or subsidence at the index operative level requiring reoperation in either cohort at a mean of 5.4 years of follow-up.

We reported two cases of reoperation in each of the ACDF groups (1.2% re-operation rate) and the TDR group (2.2%) (Figure 1). Various studies have reported radiological adjacent segment degeneration as defined by a change in Kellgren-Lawrence grading at adjacent levels [17,18]. It should be noted, however, that radiological deterioration may not be associated with pain or an increased incidence of reoperation and may even have limited clinical significance. We did not find a statistically significant increase in rates of reoperation between the two groups after a mean of 5.4 years of follow-up.

There were some limitations in the methodology of this study. ACDF surgery and TDR surgery were not randomly allocated. This largely depended on surgical preference, and while both groups were found to be equivalent in terms of age and gender, this theoretically risked potential allocation bias. Despite the excellent rate of follow-up in both groups, the failure to obtain 100% follow-up introduces the potential for a small recall bias (where those patients who did not reach final follow-up could be statistically different from those that did).

This study assessed radiographic fusion but did not assess changes in other radiographic parameters such as segmental motion, which some studies have suggested may be associated with neck pain and range of motion [18]. This could be a topic for future study. Further work could also focus on the correlation of radiological ASD with clinical symptoms and reoperation, as well as the biomechanics of ASD and whether force transmission at adjacent levels and the risk of developing ASD are equivalent regardless of the index operative level.

## Conclusions

Our findings are consistent with the results of previous work suggesting that both of these procedures lead to clinically significant improvements in pain and function scores. We were not able to find a statistically significant difference in the clinical outcomes as measured by the NDI, the VAS scores of the neck and arm, the overall complication rate, or the rates of reoperation at a mean average of over 60 months of follow-up. Despite previous literature suggesting increased rates of ASD after ACDF when compared with TDR, we did not find that this correlated to clinical outcomes or rates of re-operation in our cohort.

The results of this study are in agreement with others' assessments of these two treatment modalities. We feel that our conclusions supplement the body of knowledge about these operative options for degenerative disc disease of the cervical spine and are a useful addition to the armamentarium of any spinal specialist in the assessment of patients with degenerative pathology of the cervical spine. 
